# Mutation study of Spanish patients with Hereditary Hemorrhagic Telangiectasia

**DOI:** 10.1186/1471-2350-9-75

**Published:** 2008-08-01

**Authors:** Ana Fontalba, Africa Fernandez-L, Eva García-Alegria, Virginia Albiñana, Eva M Garrido-Martin, Francisco J Blanco, Roberto Zarrabeitia, Alfonso Perez-Molino, Maria E Bernabeu-Herrero, Maria-Luisa Ojeda, Jose L Fernandez-Luna, Carmelo Bernabeu, Luisa M Botella

**Affiliations:** 1 Centro de Investigaciones Biologicas, CSIC, Ramiro de Maeztu, 9. Madrid 28040, Spain; 2 Departamento de Genetica Medica. Hospital Marques de Valdecilla. Santander. Spain; 3 Departamento de Medicina Interna, Hospital Sierrallana, Torrelavega, Santander, Spain; 4 Centro de Investigación Biomédica en Red de Enfermedades Raras (CIBERER), Instituto de Salud Carlos III (ISCIII), Madrid, Spain

## Abstract

**Background:**

Hereditary Hemorrhagic Telangiectasia (HHT) is an autosomal dominant and age-dependent vascular disorder characterised mainly by mutations in the Endoglin (ENG) or activin receptor-like kinase-1 (ALK1, ACVRL1) genes.

**Methods:**

Here, we have identified 22 ALK1 mutations and 15 ENG mutations, many of which had not previously been reported, in independent Spanish families afflicted with HHT.

**Results:**

We identified mutations in thirty-seven unrelated families. A detailed analysis of clinical symptoms was recorded for each patient analyzed, with a higher significant presence of pulmonary arteriovenous malformations (PAVM) in HHT1 patients over HHT2. Twenty-two mutations in ALK1 and fifteen in ENG genes were identified. Many of them, almost half, represented new mutations in ALK1 and in ENG. Missense mutations in ENG and ALK1 were localized in a tridimensional protein structure model.

**Conclusion:**

Overall, ALK1 mutations (HHT2) were predominant over ENG mutations (HHT1) in our Spanish population, in agreement with previous data from our country and other Mediterranean countries (France, Italy), but different to Northern Europe or North America. There was a significant increase of PAVM associated with HHT1 over HHT2 in these families.

## Background

Hereditary Hemorrhagic Telangiectasia (HHT) (or Osler-Weber-Rendu syndrome is an autosomal and age-dependent vascular disorder (MIM #187300) [1,2] diagnosed according to clinical criteria [3]: epistaxis, telangiectases located in specific sites on the skin, and visceral involvement, including arteriovenous malformations (AVMs) in lung, brain and liver or telangiectases in the gastrointestinal tract. These features, together with the dominant inheritance, constitute the Curaçao criteria for the diagnosis of the disease, being positive if 3 out of the 4 criteria are present in a patient.

Two main loci have been identified as the genes responsible for about 85% of the cases in the HHT disease: endoglin (*ENG*; 9q34) [4] mutated in HHT type 1 (MIM #131195) and the activin receptor-like kinase-1 (*ALK1 *or *ACVRL1*; 12q13) [5] affected in HHT type 2 (MIM #601284). Recently, a third and a fourth loci for HHT have been mapped to chromosomes 5 and 7 respectively, but the specific genes have not yet been identified [6,7]. In addition, the juvenile polyposis and hereditary haemorrhagic telangiectasia (JPHT), caused by *MADH4 *mutations [8] and the primary pulmonary hypertension [9] caused by BMPRII or ALK1 mutations are related diseases showing clinical symptoms of HHT. Noteworthy in all cases, members of the TGF-β system are involved, including signalling receptors (ALK1, BMPRII), auxiliary receptors (ENG) and transcriptional co-activators (Smad4).

TGF-β signals upon binding to type II receptor on the surface of the cell. Once bound by TGF-β, type II receptor recruits and phosphorylates type I receptor. Both TGF-β receptors type I and II are serine/threonine kinases, and the activated type I receptor phosphorylates the Smad family of coactivators [10]. Endoglin interacts and cooperates with ALK1 in the TGF-β signalling pathway of endothelial cells [11,12]. Recently it has been reported that BMP9 and BMP10 are functional activators of ALK1 [13].

Protein expression studies in human umbilical vein endothelial cells and peripheral blood activated monocytes have proposed haploinsufficiency as the most likely model explaining HHT1, since most mutated endoglin alleles are not expressed on the cell surface [14-16]. Similarly, haploinsufficiency may account for HHT2, although the abundance of missense mutations affecting exons 7 and 8 suggests a misfunctioning of certain mutated ALK1 proteins [17].

To date, more than 500 mutants have been reported in HHT Mutation database, in both *ALK1 *and *ENG *genes [18]. Mutations in *ALK1 *are spread all over the 9 translated exons (from 2 to 10), whereas *ENG *mutations are found in the 12 exons coding for the extracellular domain and no mutant has ever been found in either transmembrane or cytoplasmic endoglin coding exons. Here, we have identified 22 *ALK1 *mutations and 15 *ENG *mutations, many of which had not previously been reported, as origins of the HHT disease in independent Spanish families.

## Methods

### Patient Samples, DNA Sequencing

Experimental research reported in this manuscript was performed with the approval of the appropriate ethics committee. Research was in compliance with the Helsinki Declaration. Informed consent was obtained from all the individuals participating in the study. They were sent to the unit for screening purposes and most of them without symptoms apart from epistaxis and the presence of mucocutaneous telangiectasias. Positive diagnoses of HHT were based on Curaçao established criteria. A code was established for each patient consisting in: family number, number of member within the family, and the year of sample reception. Genomic DNA was isolated from peripheral blood using the Qia-amp Mini kit (Qiagen, Germany). Mutations were analyzed by direct sequencing of amplified exons for *ALK1 *and *ENG*. The fifteen *ENG *exons and the nine *ALK1 *exons were amplified by PCR using the HotMaster Polymerase (Eppendorf, Germany) and sequenced using a cycle sequencing protocol (Applied Biosystems, USA) with previously reported primers [13,18,19]. ALK1 cDNA sequence derives from the GenBank reference sequence: 4557242 (NM_000020.1). ENG cDNA sequence derives from the GenBank reference sequence: 33871100 (BC014271.2). All the new variants described in the present paper are deposited at the Public HHT Mutation Database (HHt.org). Nucleotides are numbered with c.1 corresponding to the A of the ATG translation initiation codon in the reference sequence.

Clinical screening was performed for all the patients in the same way concerning lung (Contrast echocardiography and thorax angio CT), liver (abdominal ultrasound and angio CT) and brain (angio-MRI), regardless of their clinical status. Thus in Tables 1 and 2 the clinical findings are shown together with mutations.

## Results

### Clinical and molecular data of HHT1 and HHT2 Spanish families

Tables 1 and 2 summarise the HHT families studied with their clinical features and identified mutations. In tables, in clinical findings, E states for epistaxis, T for cutaneous telangiectasia and, PAVM, HAVM and CAVM for pulmonary, hepatic and cerebral arterio-venous malformations, respectively. GB is gastrointestinal bleeding.

Every DNA sample from clinically affected members of each family was screened for mutations in *ENG *or *ALK1 *genes without previous linkage analysis. When new missense variants were found in one single affected member, we made sure that the mutation was not a polymorphism by sequencing 100 healthy individuals, including relatives of the patient.

### Endoglin mutations

Fifteen unrelated families had 13 different mutations consisting in nonsense, missense, short deletion/insertion or intronic mutations in ENG as shown in Additional file 1.

#### Exon 1

**Family 80**, with one affected member showed the missense change, c.14C>T; p.T5M, considered as a variant [19]. Up to the present moment, we have not found any other mutation responsible for the phenotype in this patient. We are trying to get consent for more affected relatives to establish the correlation "variant"/phenotype, and we are re-screening the DNA for the presence of new mutations.

#### Exon 2

**Family 54 **with one patient showing HAVM and PAVM and a non-described deletion in exon 2, spanning 14 bp across the exon 2-intron 2 boundary (c.212_219+6 del 14) according to HUGO (Hierarchical Union of Genes from Operons) recommendations. The latter corresponds to the six first nucleotides after exon 2. The deletion in exon 2 is leading to a frameshift from aminoacid 71.

#### Exon 4

**Family 51 **was screened in one affected member showing the missense mutation, c.392C>T; P131L [20]. This patient showed PAVM and HAVM in addition to gastric bleeding. Although some studies in literature consider it a polymorphism and has been found as homozygous variant in unaffected subjects, we have found this mutation in two different families, one previously reported by us with 3 affected members and an exact correlation genotype/phenotype. In this particular family 51, other members without symptoms did not present the mutation.

#### Exon 5

**Family 30 **with three patients showing the new missense mutation, c.646A>G; p.K216Q, One of the three affected members had a PAVM and HAVMs, and had suffered from a brain abscess. Another patient had PAVMs.

#### Exon 6

**Family 49 **had a clinically diagnosed patient showing a duplication c.771dupC [20] leading to a frameshift from the 258 amino acid. The affected member showed PAVM and CAVM. **Family 42 **represented by one affected member showing the novel missense mutation c.812T>A; p.I271N.

#### Exon 9a

**Family 61 **with one affected member showed the mutation, c.1169G>A leading to a nonsense change, p.W390X, not described so far.

#### Exon 9b

**Family 67 **with one affected member, showing the missense mutation c.1274C>G leading to the change p.A425G. This patient had a PAVM.

#### Exon 10

**Family 45 **with three affected relatives, showing the nonsense mutation c.1365C>T; p.Y455X. One of the affected members had gastric bleeding and another a PAVM. This mutation had previously been found by Westwoood and Warner (2007; HHT Foundation database).

**Family 73 **with relatives in Portugal and Spain, harbored the nonsense mutation (c.1414C>T) leading to p.Q472X [21]. Two patients had GB and another PAVM.

### Intron 11

A total of 5 different families showed mutations in this intron, 3 of them having the same type of variant, c.1686+5G>C, in spite of being independent families. This mutation was present in affected members of families number 3, 31 and 33.

In **Family 3**, four members were screened. Two patients were affected of PAVMs: one of them suffered from a brain abscess in addition of PAVM, a third member had CAVMs, and the fourth showed CAVMs and HAVMs.

A third unrelated family, **Family 31**, had the same change with a member suffering from PAVMs.

**Family 33 **with 2 affected members; one of the patients showed PAVM and HAVM, and the other HAVM.

In **Family 39 **the mutation c.1686+1delG, was present in one affected member. This member had gastric bleeding and a PAVM. None of the three intron 11 mutations had been previously published.

In **Family 65 **three affected members had the change, c.1689+3A>G, two of them with PAVMs.

### ALK1 mutations

A total of 22 independent families were found as carriers of ALK1 mutations. These mutations were distributed over 8 ALK1 coding exons, from 3 to 10. In addition, intronic mutations in introns 4, 6, 7 and 9 have been found Additional file 2

### Exon 3

**Family 34 **showed a missense mutation, c.107G>A, p.C36Y [22].

**Family 74 **represented by one patient had a deletion in exon 3, c.144-145insG, mutation previously described [23] leading to the frameshift, nonsense p.A49fs. The patient had HAVM.

### Exon 4

**Family 53 **presented a duplication c.353_360dupAGCTGGCC leading to a frameshift mutation, p.L121fs not previously described, in two affected members, one of them with PAVM.

### Intron 4

**Family 35 **had the change c.525+1G>A in intron 4, detected in two members, both had PAVMs and one of them in addition showed HAVM [27].

### Exon 5

**Family 41 **showed a c.567delG deletion in one patient leading to a p.G189fs new frameshift mutation.

### Exon 6

**Family 52 **showed a nonsense new mutation, c.663G>A, p.W221X, in two affected members with HAVMs.

**Family 59 **showed the mutation c.673_674delAG deletion leading to a frameshift change p.S225fs, not previously described. The patient showed HAVMs.

**Family 69 **showed the same mutation of family 59, c.673_674delAG leading to a frameshift p.S225fs. The patient showed HAVMs.

### Intron 6

**Family 29 **had in -2 position of intron 6 the mutation c.773-2A>G [28].

### Exon 7

**Family 56 **had two affected members with an ATGCGGC duplication in exon 7 leading to a new p.L310 frameshift. One member had HAVMs, while the other, 12 years old, had no obvious symptoms.

**Family 103 **had an affected member with the mutation, c.988G>T, a missense change p.D330Y [23].

**Family 97 **had a patient with the missense mutation, c.1030T>C, resulting in a change **Intron 7**

**Family 50 **had the change c.1048+5G>T in intron 7 detected in 3 members, one affected of PAVMs and the others (under 219 without clinical manifestations.

### Exon 8

**Family 57 **had an affected member with the missense mutation, c.1135G>A leading to p.E379K [20] and HAVMs.

**Family 44 **with four patients showing the mutation, c.1205G>A, leading to the missense change p.G402D not reported before. Three of these patients had also PAVMs.

**Family 46 **had 3 affected relatives with the missense mutation, c.1232G>A leading to p.R411Q [24], two of them had PAVMs and one in addition HAVMs.

**Family 60 **had the missense mutation, c.1121G>A; p.R374Q [17] and the affected member had PAVMs and HAVMs, as well as gastric bleeding.

### Exon 9

**Family 38 **had three members with a new missense mutation, c.1261T>G; p.Y421D. One of them had PAVM.

**Family 55 **has a missense change, c.1345C>A; p. P449T previously described [25]. The patient had PAVM, HAVM and GB.

### Intron 9

**Family 62 **had the new mutation c.1378-1G>T, involving intron 9. The mutation was found in two affected members.

### Exon 10

**Family 77 **harbored the nonsense change c. 1435C>T; p.R479EX [7] in two patients.

**Family 36 **had 2 members with the missense change c.1405C>T; p.R484W, [26] one of them had an aortic fistula at birth.

**Figure 1:**
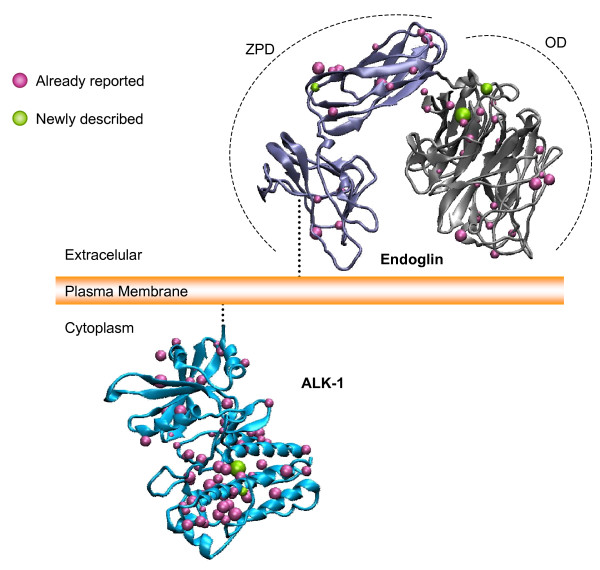
The missense mutations previously described for the extracellular part of endoglin and the intracellular part of ALK1 are shown as blue spheres. The new mutations described in this report for both proteins appear as green spheres. The volume of the spheres is exclusively related to the size of the mutated residue side chain.

Among all the ENG and ALK1 mutations, missense mutations are predicted to have an important impact on protein structure. Figure 1 shows the distribution in theoretical 3D models of all the missense mutations including the newly described in this work, for both, endoglin and ALK1 proteins. Endoglin structure was obtained according to [29] and is a theoretical ab initio model. The theoretical ALK1 structure was obtained by homology according to the procedure described [17]. Endoglin model is made for the extracellular domain, while ALK1 model represents the cytoplasmic domain. Overall, there is a predominant allocation of missense mutations in the orphan domain (OD) of Endoglin. Two out of three missense new mutations reported for Endoglin in this paper fall in the OD, while the third one is located in the Zona Pellucida Domain (ZPD), according to the prediction [29].

In ALK1, most of the missense mutations, including the newly described ones here, are clustered around the pockets of the kinase domain as described [17].

## Discussion

The first paper on mutation analysis in Spanish HHT patients reported results on 17 families and now on 37 different families. All patients are familiar cases with no sporadic case found in such cohort. We are presenting the results obtained in a total of 74 clinically diagnosed patients distributed among 37 independent families with identified mutation. In addition, 15 more patients belonging to 11 families were clinically screened, but the data are not presented in this study, since the corresponding mutation analysis is not yet finished.

The actual rate of success in molecular diagnosis is around 75% (37 out of 49). In order to increase this rate, we are currently using additional approaches like sequencing SMAD 4 locus and MLPA (analysis of whole exonic deletion or duplication mediated by ligation).

Out of the mutations, 15 were in *ENG *and 22 in *ALK1*. Thus, in Spain, HHT due to the fact that mutation in *ALK1 *is higher than in *ENG*, following a similar trend (6 vs 11) as in our previous studies [22]. This is in agreement with the data from other mediterranean populations like France [30] and Italy [31] but different from Canada, USA [18,24] and Northern Europe populations [32] where pathogenetic mutations in *ENG *are more frequent than in *ALK1*.

Although the number of patients included in this study is a bit too small, still we have taken advantage of drawing some phenotype/genotype correlation features. Concerning the clinical symptoms, when comparing Tables 1 and 2, the number of PAVMs was more frequent (55.1%) in HHT1 than in HHT2 (33.3%). The slightly higher percentage of PAVMs found among the HHT2 patients versus previous reports may be explained by the highly sensitive screening method used, contrast echocardiography with injected bubbles, which can detect even small PAVMs. Gastrointestinal bleeding (GB) and CAVMs were also more frequent in HHT1 (13.8% and 10%, respectively) than in HHT2 (5.5% and none, respectively). Finally, HAVMs were slightly more frequent in HHT2 (36.1%) than in HHT1 (34.4%). The same protocol of screening (for lung, liver, brain) was applied for all patients regardless of the clinical status. These data are compatible with previous reports on genotype phenotype correlations [33-35], especially with the last which contains a large list of patients from the French-North Italian network [34]. It is also important to stress the fact that the severity of symptoms, especially the amount of epistaxis and frequency increased tremendously in age, as HHT signs are age-dependent. It is interesting at this point to consider intronic mutations, and the age-dependence for the genotype/phenotype correlation, the latter will depend on the rate of correct splicing from the wild type allele, versus the aberrant one due to mutation. The splicing machinery action maybe age-dependent, as seen in family 50, where the father had symptoms while his two sons remained still without clinical criteria.

Many of the mutations found are new; 8 in *ALK1 *and 8 in *ENG*. It is also noteworthy the finding of 3 independent families harboring the same mutation in intron 11 of endoglin, a newly described mutation (c.1686+5G>C). This may represent a hot spot mutation according to our series data in Spanish population. Alternatively a single mutational event could take place in a common ancestor of the three families which are now apparently unrelated, then representing a founder effect in the Spanish population.

The mutations found in *ENG *and *ALK1 *are spread all over the different exons and some of them were intronic. In *ENG*, along exons 1(1), 2(1), 4 (2), 5(1), 6 (2), 9a (1), 9b (1) and 11(1), as well as intron 11(3). In *ALK1*, along exons 3 (2), 4 (1), 5 (1), 6 (2), 7 (3), 8 (4), 9 (2) and 10 (2), as well as introns 2 (1), 4 (1), 6 (1), 7(1) and 9 (1).

Haploinsufficiency is the commonly accepted pathogenic model especially for HHT1 (17). It has been shown that missense endoglin mutants are not found expressed on the cell surface [14,16,22] suggesting that these missense mutations have an impact on the protein structure. Interestingly, there is a slightly predominant allocation of missense pathogenic mutations in the OD of endoglin (Figure 1, top), although other mutations are also present in the ZPD. This argues in favour of an important role of OD and ZPD in the endoglin post-translational modifications, folding and export to the outside of the membrane. In the case of ALK1, most missense mutations involve the kinase domains. In the latter case, a less active kinase may be the origin of the disease, rather than a complete loss of half of the protein (haploinsufficiency). In fact, in [22] we have shown that some ALK1 missense mutants are expressed on the cell surface. When comparing the ALK1 missense versus nonsense or frameshift mutations (null alleles), we have not found correlations in terms of predominant clinical symptoms in one of the classes. Out of 22 ALK1 families, 11 had missense and 11 had nonsense mutations, the frequency of internal arteriovenous malformations was exactly the same, 8/11 in both classes, suggesting that sometimes missense alleles may not be functional, or even deleterious.

## Conclusion

- A series of 37 mutations have been found as origins of HHT, both type 1 and 2 in Spanish families

- Out of 37, almost half of them were newly described mutations.

- There is a predominance of mutations in *ALK1 *(22) versus 15 in *Endoglin *as it is current for other mediterranean countries

- We see a predominance of pulmonary arteriovenous malformations in HHT1 (52% of the screened patients) versus HHT2, whereas in the latter the hepatic arterio venous malformations is slighty more prevalent.

- Missense mutations in *ALK1 *cluster predominantly in kinase domains of exons 7 and 8, whereas the less abundant missense mutations found in *Endoglin *are scattered in the extracellular domain of the protein.

## Competing interests

The authors declare that they have no competing interests.

## Authors' contributions

All the authors have read and approved the final manuscript. A large part of the mutational analysis was done by AF, EGA under the supervision of JLFL. The rest of the mutational analysis was done by AFL, VA, EMGM, MLO, MEBH completing the analysis of mutations. Bank data collection, writing of the manuscript and the direction of all the work was done under LMB, the main author. CB shared some aspects of the direction together with LMB, and the clinicians RZ and APM screened for the patients. FJB contributed with the 3D model.

## Pre-publication history

The pre-publication history for this paper can be accessed here:



## Supplementary Material

Additional file 1 Table 2. Click here for file

Additional file 2 Table 2. Click here for file
